# Overexpression of TMEM150A in glioblastoma multiforme patients correlated with dismal prognoses and compromised immune statuses

**DOI:** 10.1371/journal.pone.0294144

**Published:** 2023-12-06

**Authors:** Si-Tong Fan, Hao-Qiang Xu, Yang He, Ming-Xiang Tu, Ke Shi, Yun-Qiang Zhang, Qiang Guo, Wen-Qiong Yang, Yong Qin

**Affiliations:** 1 Department of Infectious Disease, Beilun District People’s Hospital of Ningbo, Ningbo City, China; 2 Department of Neurology, Sinopharm Dongfeng General Hospital, Hubei University of Medicine, Shiyan City, China; 3 Department of Neurosurgery, Sinopharm Dongfeng General Hospital, Hubei University of Medicine, Shiyan City, China; 4 Department of Neurology, Yunyang District People’s Hospital, Shiyan City, China; 5 Department of Thoracic Surgery, Beilun District People’s Hospital of Ningbo, Ningbo City, China; 6 Department of Cardiothoracic Surgery, Taihe Hospital, Hubei Medical University, Shiyan City, China; 7 Department of Neurology, Shenzhen Lansheng Brain Hospital, Shenzhen City, China; All India Institute of Medical Sciences, INDIA

## Abstract

Transmembrane proteins have exhibited a significant correlation with glioblastoma multiforme (GBM). The current study elucidates the roles of transmembrane protein 150A (*TMEM150A*) in GBM. Data on patients with GBM were collected from The Cancer Genome Atlas and Xena databases. The objective was to identify the expression levels of *TMEM150A* in patients with GBM, and evaluate its diagnostic and prognostic values, accomplished using the receiver operating characteristic and survival analyses. On a cellular level, Cell Counting Kit-8, Wound healing, and Transwell experiments were performed to gauge the impact of *TMEM150A* on cell growth and migration. The study further investigated the correlation between *TMEM150A* expression and immune status, along with ribonucleic acid (RNA) modifications in GBM. The findings demonstrated *TMEM150A* overexpression in the cancerous tissues of patients with GBM, with an area under the curve value of 0.95. *TMEM150A* overexpression was significantly correlated with poor prognostic indicators. *TMEM150A* overexpression and isocitrate dehydrogenase (IDH) mutation status were predictive of poor survival time among patients with GBM. *In vitro* experiments indicated that suppressing TMEM150A expression could inhibit GBM cell proliferation, migration, and invasion. Moreover, *TMEM150A* overexpression was associated with stromal, immune, and estimate scores, immune cells (such as the T helper (Th) 17 cells, Th2 cells, and regulatory T cells), cell markers, and RNA modifications. Therefore, *TMEM150A* overexpression might serve as a promising biomarker for predicting poor prognosis in patients with GBM. Inhibiting *TMEM150A* expression holds the potential for improving the survival time of patients with GBM.

## Introduction and preliminaries

Glioblastoma multiforme (GBM) is a highly malignant astrocytoma with limited treatment options. Currently, the preferred approaches for managing GBM involve surgical resection of tumour tissues followed by radiotherapy and chemotherapy. However, the prognosis of patients with GBM patients is significantly poor, with less than 6% of patients surviving beyond 5 years [1]. Improving the survival rate for patients with GBM remains a pressing concern demanding resolution. In recent years, several biomarkers have been identified for their pivotal roles in GBM progression [2–4]. For instance, Shen et al. reported cardiotrophin-like cytokine factor 1 (*CLCF1*) overexpression in GBM tissues, which was associated with an unfavourable prognosis. Inhibiting *CLCF1* expression significantly reduced cell proliferation and migration, induced apoptosis, and prompted cell cycle arrest in GBM [4]. These findings demonstrate the need for identifying new biomarkers to predict the survival time of patients with GBM and improve the overall prognosis of patients with cancer.

Transmembrane proteins serve as channels that control the entry and exit of various substances across cellular membranes. Several investigations have linked transmembrane proteins with cancer [5–8]. For instance, in non-small cell lung cancer (NSCLC), transmembrane protein (TMEM) 229A (*TMEM229A*) is significantly underexpressed, and this underexpression is associated with poor prognosis in patients with cancer. Conversely, *TMEM229A* overexpression can inhibit NSCLC cell proliferation, migration, and invasion while promoting E-cadherin overexpression and N-cadherin and matrix metalloproteinase 2 underexpression [8]. Similarly, *TMEM168* is overexpressed in GBM and is associated with shorter survival in patients with GBM. Inhibiting *TMEM168* expression can impede the viability of GBM cancer cells, induce cell cycle arrest, and enhance apoptosis, which are mechanisms associated with the obstruction of the WNT/β-catenin pathway [7]. Another member of the transmembrane protein family, *TMEM150A*, plays a role in cell homeostasis by regulating cytokine secretion and transcription. Decreased *TMEM150A* expression could result in increased lipopolysaccharide (LPS)-induced cytokine secretion [9]. Furthermore, the relationship between ribonucleic acid (RNA) modifications and GBM progression was inseparable [10–12]. For instance, long-chain non-coding RNA LINC00839 was overexpressed in glioma stem cells (GSCs). LINC00839 overexpression was strongly associated with GBM progression and radiation resistance. Methyltransferase-like protein (METTL) 3 could mediate the m6A-modified LINC00839 activation of WNT/β-catenin signalling, thereby enhancing tumour progression and radiation resistance [10]. In addition, a significant correlation exists between the immune microenvironment and GBM progression [13]. Therefore, this study aims to analyse the role of *TMEM150A* in GBM progression, alongside assessing the relationship between *TMEM150A* and the immune microenvironment and RNA modifications to provide a new candidate molecule for treating patients with GBM.

## Methods

### The cancer genome atlas (TCGA) and Xena databases

TCGA stands as an open-access cancer database comprising transcriptional data from patients with cancer. In contrast, the Xena database comprises transcription data from normal tissues of patients. In this study, transcription data was obtained from five normal tissues and 166 cancer tissues of patients with GBM, within the confines of TCGA. In parallel, the Xena database yielded access to transcription data from 1152 normal tissues, sourced from the genome-tissue expression (GTEx) database. Furthermore, data pertaining to *TMEM150A* expression, clinicopathological features, and prognostic data of the patients with GBM were obtained from TCGA.

### The relationship between *TMEM150A* expression and the clinical features of patients with GBM

*TMEM150A* expression levels were analysed in normal and cancer tissues. Subsequently, the *TMEM150A* expression data was integrated with clinical data from patients with GBM, encompassing variables such as age, sex, tumour grade, and survival status. The clinical characteristics of the patient groups were assessed and patients were categorised based on median *TMEM150A* expression levels, comparing the differences between high- and low-*TMEM150A* expression groups.

### Identification of *TMEM150A* expression association with diagnosis and patient prognosis

Receiver operating characteristic (ROC) analysis was used to determine the diagnostic significance of *TMEM150A* in cancerous and normal tissues obtained from patients with GBM. The area under the ROC curve was used as the evaluation index. Additionally, data on *TMEM150A* expression and prognosis of patients with GBM were merged to examine the association between high- and low-*TMEM150A* expression levels and overall survival (OS) time, disease-specific survival (DSS), and progression-free interval (PFI) among patients with GBM. The patient groups were categorised based on median *TMEM150A* expression levels.

Association between *TMEM150A* expression and survival time in various subgroups of patients with GBM

*TMEM150A* expression data was combined with clinical characteristics and prognostic information from patients with cancer. This investigation was undertaken to explore the relationship between *TMEM150A* expression levels and OS, DSS, and PFI of patients with GBM across subgroups based on age, sex, race, isocitrate dehydrogenase (IDH) status, and Karnofsky performance score (KPS). Patient groups were categorised based on median *TMEM150A* expression levels.

### TMEM150A associated nomograms

Univariate Cox regression analysis was performed to assess the correlation between sex, age (≤60 or >60 years), KPS (<80 or ≥80), IDH status, *TMEM150A* expression level, and poor prognostic indicators in patients with GBM. Variables with a significance level of P<0.05 were further subjected to multivariate Cox regression analysis. Additionally, nomograms were used as a tool to evaluate the prognosis of patients with cancer [14–16]. Thus, nomograms were developed based on the results of the multivariate Cox regression analysis, enabling the prediction of prognosis among patients with GBM.

### Tumour immune estimation resource (TIMER) database

The TIMER database, an online cancer immune database, facilitates the visualisation of the correlation between gene expression and cancer immune cells, along with cellular markers. In this study, the TIMER database was used to explore *TMEM150A* expression across various cancer tissues. This aimed to elucidate the relationship between *TMEM150A* expression and tumour purity, immune cells, and immune cell markers.

### Identification of TMEM150A expression levels in relation to the tumour immune status

The single-sample Gene Set Enrichment Analysis (GSEA) and ESTIMATE methods were used to analyse the tumour microenvironment within the cancerous tissues obtained from patients with GBM sourced from TCGA. *TMEM150A* expression data and immune cell markers were extracted from these cancer tissues using the Perl programming language, and correlation analysis was performed to investigate the association between *TMEM150A* expression and the infiltration of various immune cells and specific immune markers. Patients with GBM were classified into two groups based on the median *TMEM150A* expression to further examine the relationship between *TMEM150A* expression and immune infiltration.

### Gene expression profiling interactive analysis (GEPIA) database

The GEPIA database comprised tissue samples from patients with cancer and healthy individuals obtained from TCGA and GTEx databases. This study used the GEPIA database to establish the correlation between *TMEM150A* expression and poor prognosis in patients with GBM.

### GSEA

Using the CAMOIP database, the cancerous tissues obtained from patients with GBM in TCGA were subjected to GSEA [17]. Additionally, GSEA could elucidate the functions and signalling mechanisms of genes [18, 19]. Therefore, GSEA of the CAMOIP database was performed to investigate the signalling mechanisms of high and low expressions of *TMEM150A*, with a significance threshold set at P < 0.05.

### Identification of the relationship between *TMEM150A* expression and RNA modifications

The RM2TARGET database systematically collected relevant literature and publicly available RNA modification gene datasets, encompassing informative target gene associations and annotations such as m6A, m1A, m5C, and m7G modifications for human genes. From this repository, the human target gene *TMEM150A* was specifically identified and the RM2TARGET database was used to identify its associated RNA modifications. Subsequently, using the TIMER database, the correlation between *TMEM150A* expression levels and RNA modifications was explored. This was corroborated through an examination of RNA sequencing data sourced from tumour tissues of patients with GBM in TCGA.

### Cell culture and construction of the *TMEM150A* inhibition model

GBM U118 cells were cultured in Dulbecco’s Modified Eagle Medium with 10% foetal bovine serum. Following this, the cells were transfected within a six-well plate, and cell proteins and RNA were collected after 24 h. Subsequently, the *TMEM150A* levels in the control group and the group with inhibited *TMEM150A* expression were examined using standard reverse transcription-polymerase chain reaction (RT-PCR) and Western blotting [20].

### Cell counting kit (CCK)-8

Ninety-six-well plates were arranged with cells in an optimal growth state. Following cell adhesion, a 10μL solution of CCK-8 was added, and the cells were incubated for 1 h to measure cell activity, denoting this time point as 0 h. The cell activity of the control group and the group with inhibited *TMEM150A* expression was measured and statistically analysed at 24, 48, and 72 h.

### Wound healing

The U118 cell suspension was introduced into a six-well plate, and upon cell adhesion, a linear scratch was created across the cell monolayer using the tip of a 200 μL pipette. The subsequent step involved the removal of any cellular debris. Following this, the culture medium was supplemented, and micrographs were taken to document the state. Finally, the process of cellular migration in response to the scratch was closely monitored under a microscope.

### Transwell assay

The lower compartment of the Transwell plate was filled with the culture medium, while the Transwell membranes were positioned in the upper compartment. A serum-free suspension of U118 cells was then prepared, and the Transwell apparatus was incubated at a controlled temperature to culture the cells under optimal conditions. Subsequently, the cell invasion capabilities were assessed and compared at distinct time intervals. Following this, the cells were fixed with an appropriate volume of formaldehyde. After thorough washing, staining and visualisation were performed.

### Statistical analysis

The Wilcoxon signed-rank test was used to identify differences in *TMEM150A* expression levels between normal and cancerous tissues. The t-test was used to determine the statistical significance of the growth and migration capabilities of two distinct U118 cell groups. The chi-square test was used to examine the relationship between TMEM150A levels and the clinical characteristics of cancer patients. Additionally, the diagnostic and prognostic capability of TMEM150A was explored using ROC and survival analyses. Spearman correlation analysis was performed to understand the relationship between *TMEM150A*, immune microenvironment, and RNA modifications. Statistical significance was set at P<0.05.

## Results

### *TMEM150A* overexpression was associated with age, diagnosis, and poor prognosis among patients with GBM

**Fig 1:**
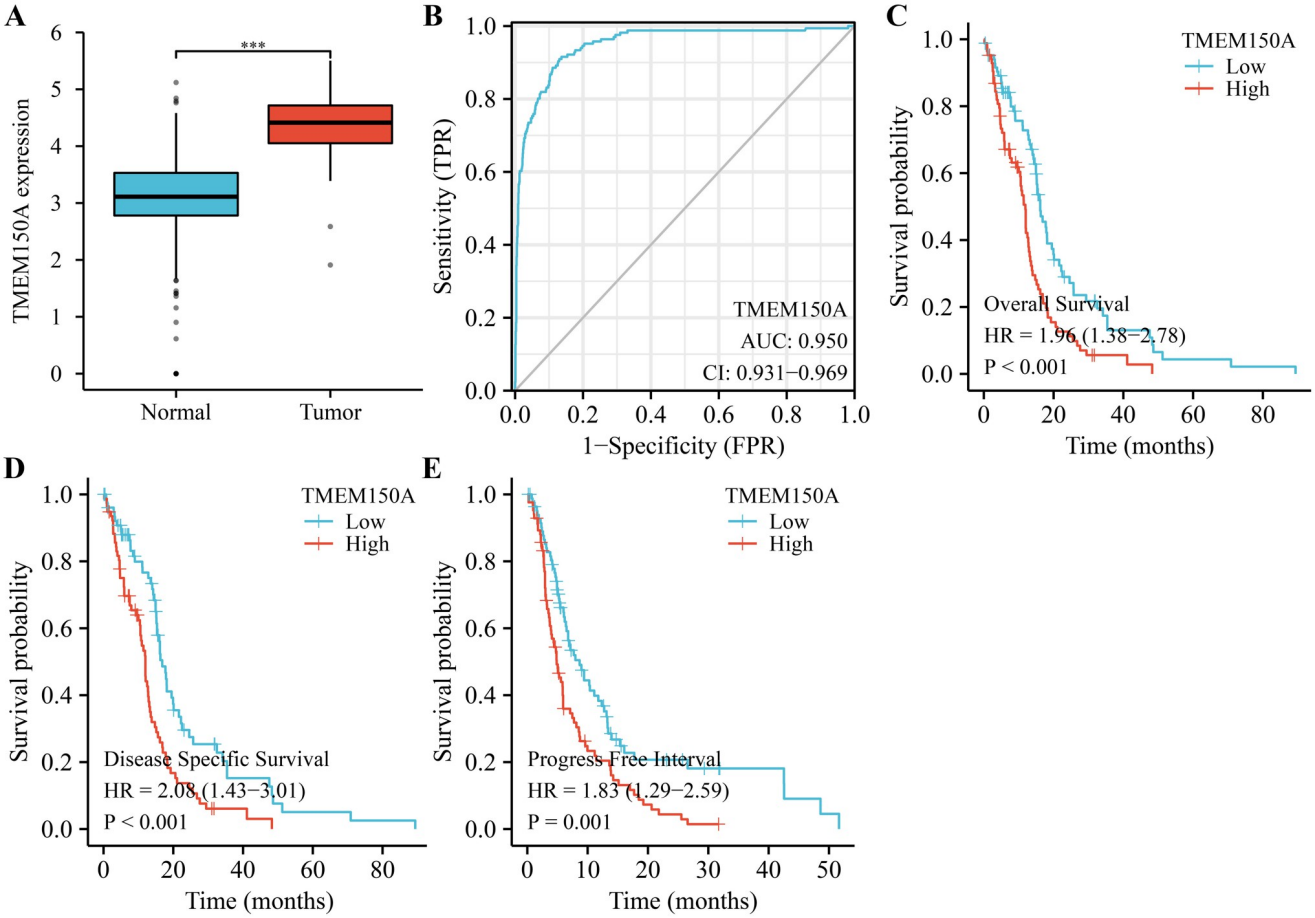
Increased TMEM150A expression level was associated with poor prognosis and diagnosis in patients with GBM. (A) TMEM150A expression in GBM from the Xena database; (B) the diagnostic value of TMEM150A using ROC analysis; (C-E) the prognostic values of TMEM150A in GBM using survival analysis. Note: TMEM, transmembrane protein; GBM, glioblastoma multiforme; ROC, operating characteristic.

**Fig 2:**
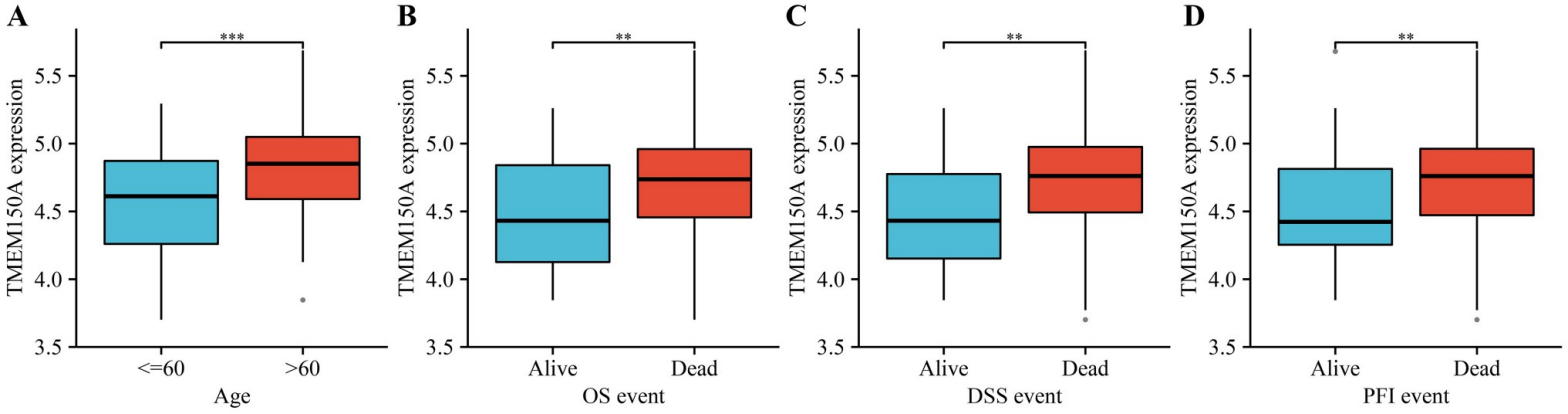
Increased TMEM150A expression level was associated with poor prognosis and age in patients with GBM. (A) Age; (B) OS event; (C) DSS event; (D) PFI event. Note: TMEM, transmembrane protein; GBM, glioblastoma multiforme; OS, overall survival; DSS, disease-specific survival; PFI, progression-free interval.

TMEM150A expression significantly increased in GBM tissues compared with normal tissues (Fig 1A). Using ROC analysis, the area under the curve for *TMEM150A* was 0.95, indicating that *TMEM150A* overexpression serves as an effective diagnostic biomarker for GBM (Fig 1B). Analysis of data from patients with GBM patient data from TCGA revealed that *TMEM150A* overexpression was correlated with poor survival rates and disease progression in patients with GBM (Fig 1C–1E). Additionally, data derived from the GEPIA database revealed that *TMEM150A* overexpression was associated with poor OS and disease-free survival (DFS) in patients with GBM (S1 Fig). Moreover, *TMEM150A* overexpression levels were associated with age, OS, DSS, and PFI in patients with GBM (Fig 2 and Table 1).

**Table 1:** The relationship between TMEM150A levels and clinical indices in patients with GBM.

Characteristic	TMEM150A		P
	Low expression	High expression	
N	84	84	
Gender			0.196
Female	34 (20.2%)	25 (14.9%)	
Male	50 (29.8%)	59 (35.1%)	
Race			0.925
Asian	3 (1.8%)	2 (1.2%)	
Black or African American	5 (3%)	6 (3.6%)	
White	74 (44.6%)	76 (45.8%)	
Age			0.013
< = 60	52 (31%)	35 (20.8%)	
>60	32 (19%)	49 (29.2%)	
KPS			0.712
<80	20 (15.6%)	16 (12.5%)	
> = 80	46 (35.9%)	46 (35.9%)	
IDH status			0.311
WT	70 (43.5%)	79 (49.1%)	
Mut	8 (5%)	4 (2.5%)	
OS event			0.077
Alive	21 (12.5%)	11 (6.5%)	
Dead	63 (37.5%)	73 (43.5%)	
DSS event			0.013
Alive	24 (15.5%)	10 (6.5%)	
Dead	54 (34.8%)	67 (43.2%)	
PFI event			0.011
Alive	23 (13.7%)	9 (5.4%)	
Dead	61 (36.3%)	75 (44.6%)	

Note: TMEM, transmembrane protein; KPS, Karnofsky performance score; GBM, glioblastoma multiforme; OS, overall survival; DSS, disease-specific survival; PFI, progression-free interval.

### *TMEM150A* overexpression was associated with poor prognosis specifically in certain subgroups of patients with GBM

**Fig 3:**
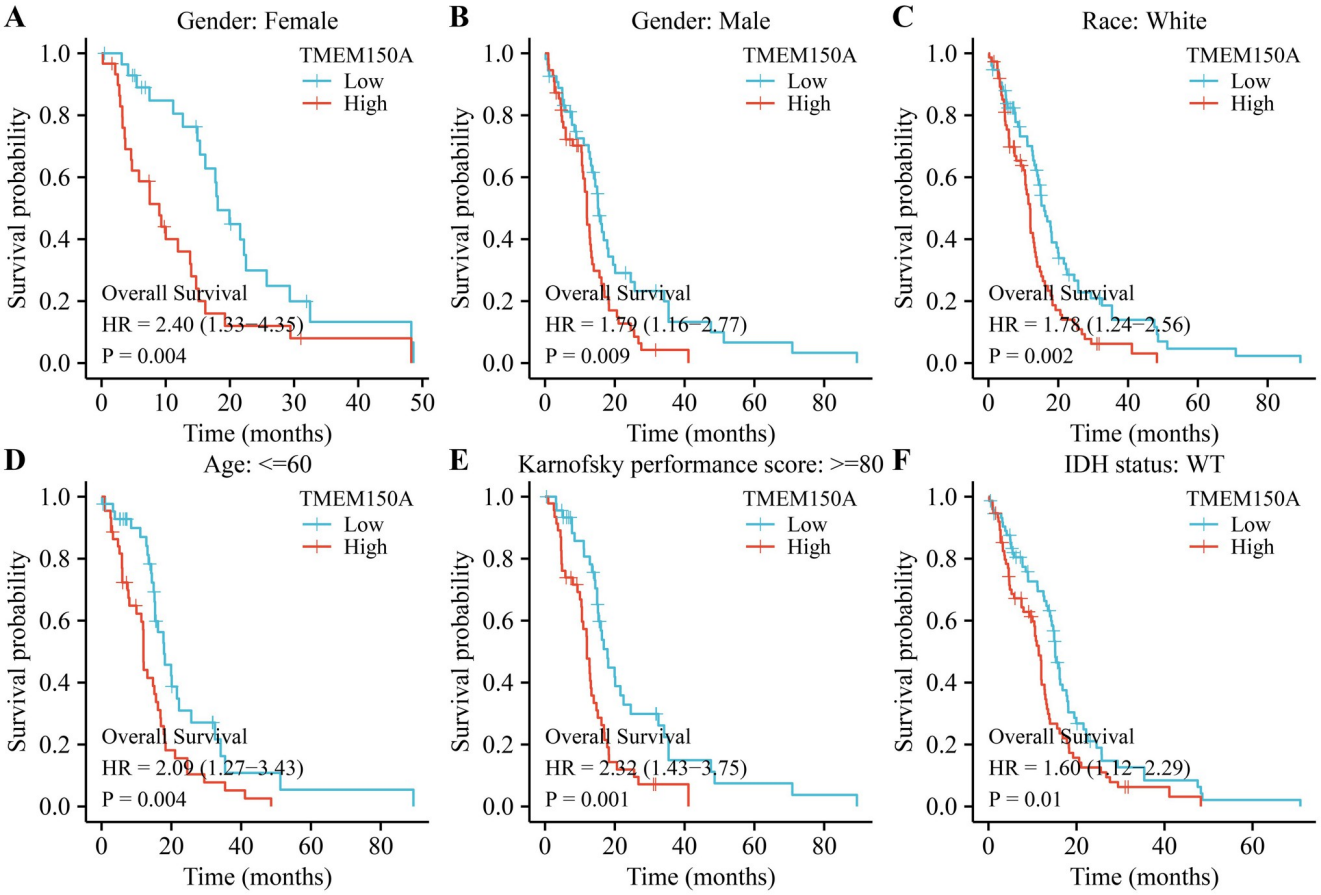
Elevated TMEM150A expression was associated with short OS in a subgroup of patients with GBM. (A) Female; (B) Male; (C) White; (D) Age ≤60; (E) KPS ≥80; (F) IDH status.Note: TMEM, transmembrane protein; GBM, glioblastoma multiforme; OS, overall survival; KPS, Karnofsky performance score; IDH, isocitrate dehydrogenase.

**Fig 4:**
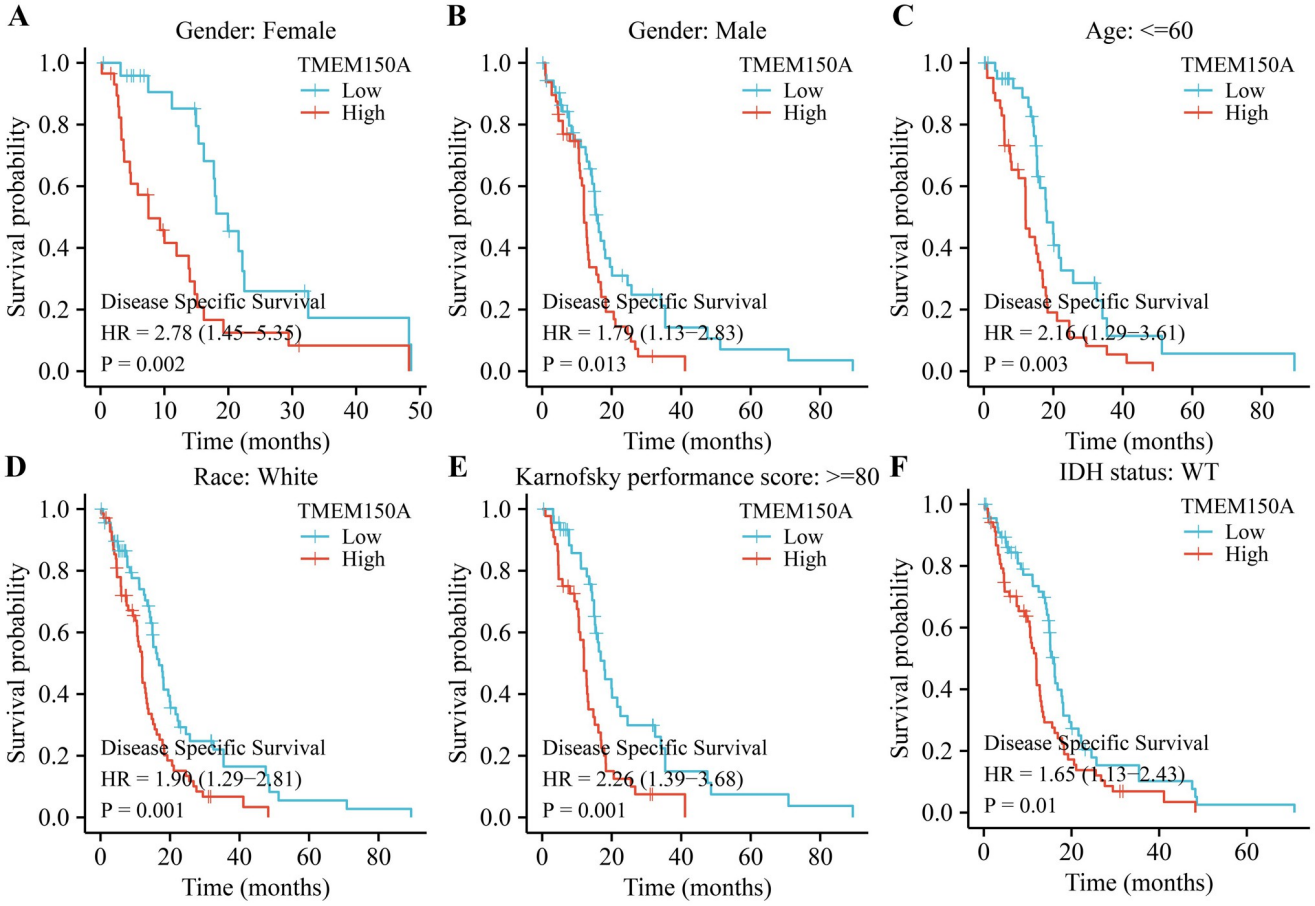
Elevated TMEM150A expression was associated with short DSS in a subgroup of patients with GBM. (A) Female; (B) Male; (C) Age ≤60; (D) White; (E) KPS ≥80; (F) IDH status. Note: TMEM, transmembrane protein; GBM, glioblastoma multiforme; DSS, disease-specific survival; KPS, Karnofsky performance score; IDH, isocitrate dehydrogenase.

**Fig 5:**
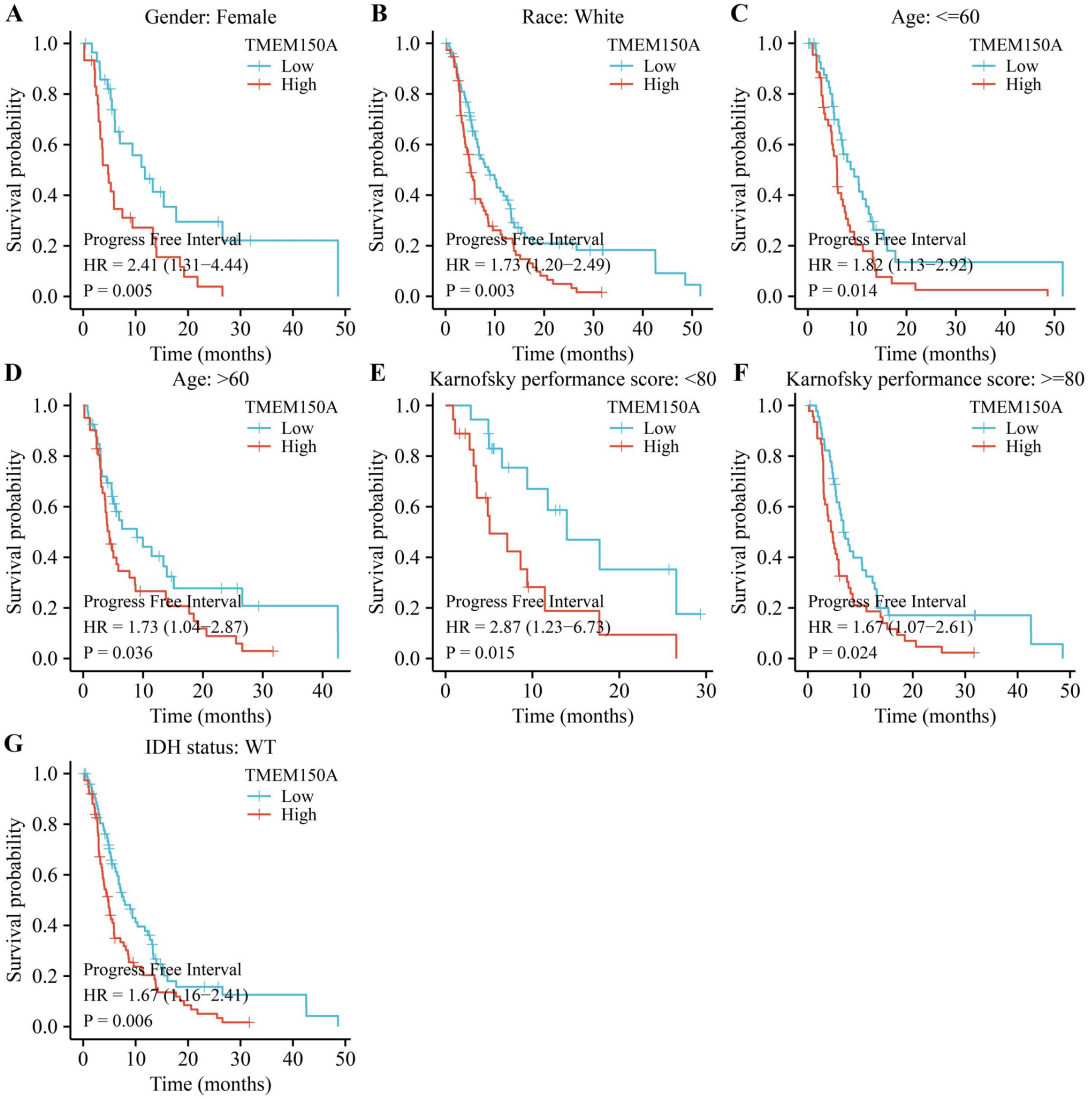
Elevated TMEM150A expression was associated with short PFI in a subgroup of patients with GBM. (A) Female; (B) White; (C) Age ≤60; (D) Age >60; (E) KPS < 80; (F) KPS ≥80; (G) IDH status. Note: TMEM, transmembrane protein; GBM, glioblastoma multiforme; PFI, progression-free interval; KPS, Karnofsky performance score; IDH, isocitrate dehydrogenase.

Survival analysis revealed that *TMEM150A* overexpression was associated with poor OS and DSS in male and female patients, regardless of their race, age ≤60, KPS≥80, or IDH wild-type (WT) status in patients with GBM (Figs 3 and 4). *TMEM150A* overexpression was associated with poor PFI in female and white patients, patients aged <60 years, patients aged >60 years, those with a KPS score of <80, those with a KPS score greater ≥80, and those with an IDH WT status, as presented in Fig 5.

### *TMEM150A* overexpression serves as an indicator of poor prognosis in patients with GBM

**Fig 6:**
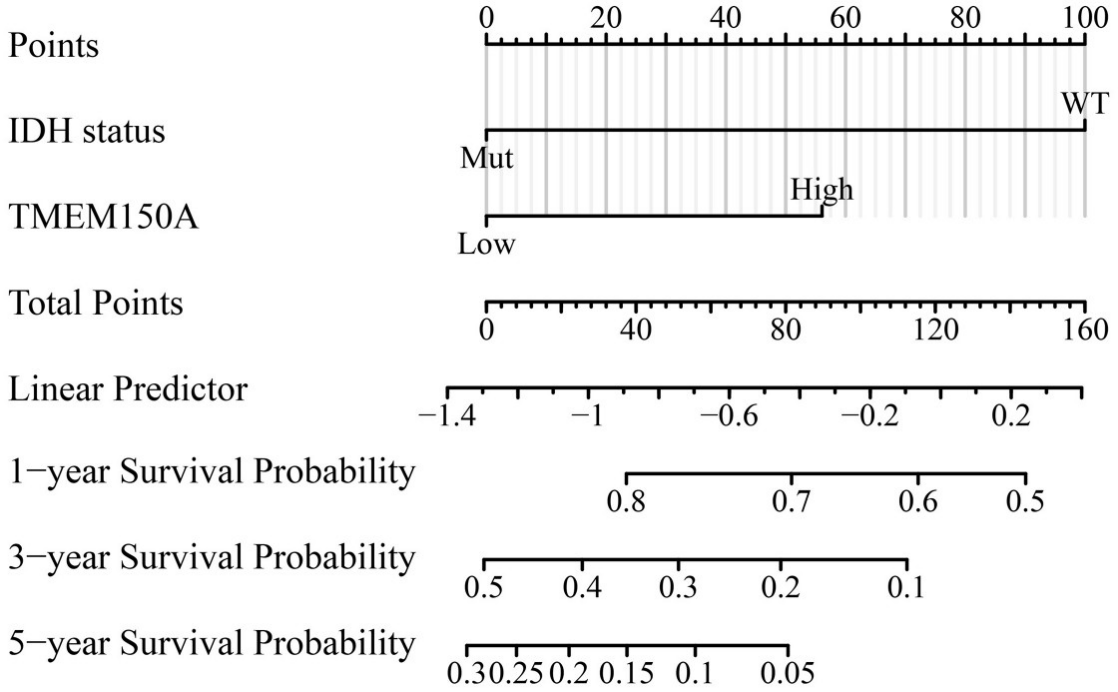
TMEM150A correlating the nomogram in terms of the OS in patients with GBM. Note: TMEM, transmembrane protein; OS, overall survival; GBM, glioblastoma multiforme.

Univariate Cox regression analysis revealed that *TMEM150A* and IDH WT status were significant predictors of poor OS, DSS, and PFI among patients with GBM, as detailed in Tables 2–4. The outcomes of multivariate Cox regression analysis further confirmed that IDH WT status and *TMEM150A* overexpression were significant predictors of poor prognosis in patients with GBM (Tables 2–4). The nomograms effectively helped visualise the prognosis and survival outcomes of patients with cancer [14–16]. Therefore, nomograms were constructed for OS, DSS, and PFI in patients with GBM based on the IDH status and *TMEM150A* expression levels (Fig 6 and S2 Fig).

**Table 2:** Cox analysis showing the factors influencing OS in patients with GBM.

Characteristics	N	Univariate analysis		Multivariate analysis	
		HR (95% CI)	P	HR (95% CI)	P
Gender	168				
Female	59	Reference			
Male	109	1.026 (0.719–1.466)	0.887		
Age	168				
< = 60	87	Reference			
>60	81	1.365 (0.973–1.915)	0.072		
KPS	128				
<80	36	Reference			
> = 80	92	0.838 (0.538–1.305)	0.434		
IDH status	161				
WT	149	Reference			
Mut	12	0.301 (0.138–0.654)	0.002	0.345 (0.158–0.756)	0.008
TMEM150A	168				
Low	84	Reference			
High	84	1.962 (1.384–2.782)	<0.001	1.813 (1.269–2.592)	0.001

Note: KPS, Karnofsky performance score; GBM, glioblastoma multiforme; OS, overall survival.

**Table 3:** Cox analysis showing the factors influencing DSS of patients with GBM.

Characteristics	N	Univariate analysis		Multivariate analysis	
		HR (95% CI)	P	HR (95% CI)	P
Gender	155				
Female	54	Reference			
Male	101	0.975 (0.667–1.425)	0.894		
Age	155				
< = 60	83	Reference			
>60	72	1.340 (0.935–1.920)	0.111		
KPS	119				
<80	29	Reference			
> = 80	90	0.919 (0.552–1.529)	0.745		
IDH status	148				
WT	136	Reference			
Mut	12	0.315 (0.144–0.686)	0.004	0.370 (0.168–0.814)	0.013
TMEM150A	155				
Low	78	Reference			
High	77	2.076 (1.429–3.014)	<0.001	1.922 (1.309–2.822)	<0.001

Note: KPS, Karnofsky performance score; GBM, glioblastoma multiforme; DSS, disease-specific survival.

**Table 4:** Cox analysis showing the factors influencing the PFI of patients with GBM.

Characteristics	N	Univariate analysis		Multivariate analysis	
		HR (95% CI)	P	HR (95% CI)	P
Gender	168				
Female	59	Reference			
Male	109	1.223 (0.855–1.750)	0.269		
Age	168				
< = 60	87	Reference			
>60	81	1.000 (0.710–1.409)	1.000		
KPS	128				
<80	36	Reference			
> = 80	92	1.544 (0.970–2.457)	0.067		
IDH status	161				
WT	149	Reference			
Mut	12	0.395 (0.183–0.852)	0.018	0.447 (0.206–0.968)	0.041
TMEM150A	168				
Low	84	Reference			
High	84	1.831 (1.294–2.590)	<0.001	1.771 (1.237–2.535)	0.002

Note: KPS, Karnofsky performance score; GBM, glioblastoma multiforme; PFI, progression-free interval.

### Decreased *TMEM150A* expression inhibited GBM cell growth and metastasis

**Fig 7:**
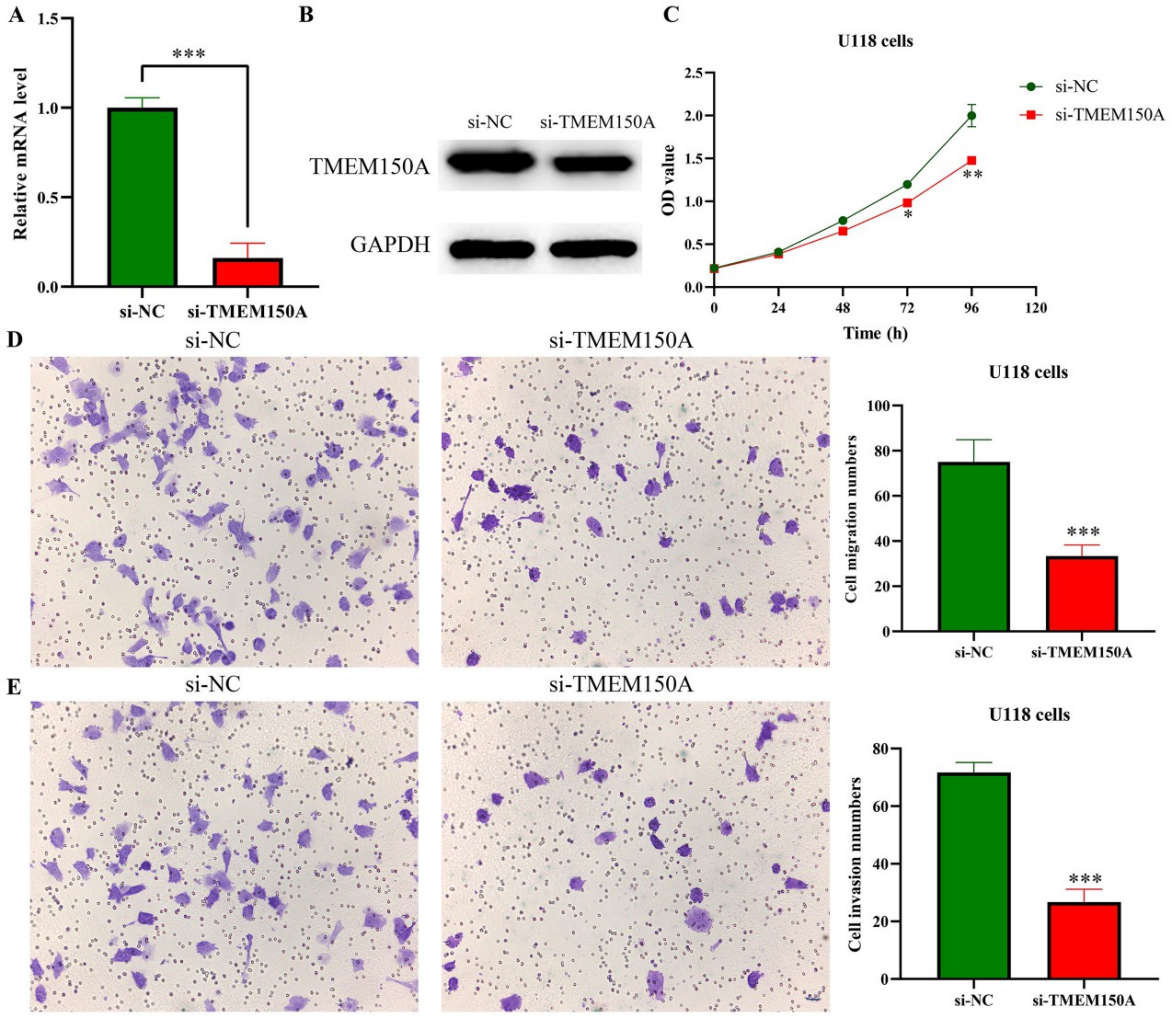
Decreased TMEM150A expression inhibits cell proliferation, migration, and invasion in GBM using CCK-8 and Transwell assays. (A-B) Successfully constructed inhibited TMEM150A expression model using RT-PCR and Western blotting; (C) cell proliferation by CCK-8; (D) cell migration by Transwell assay; (E) cell invasion by Transwell assay. Note: TMEM, transmembrane protein; GBM, glioblastoma multiforme; CCK-8, Cell Counting Kit-8.

**Fig 8:**
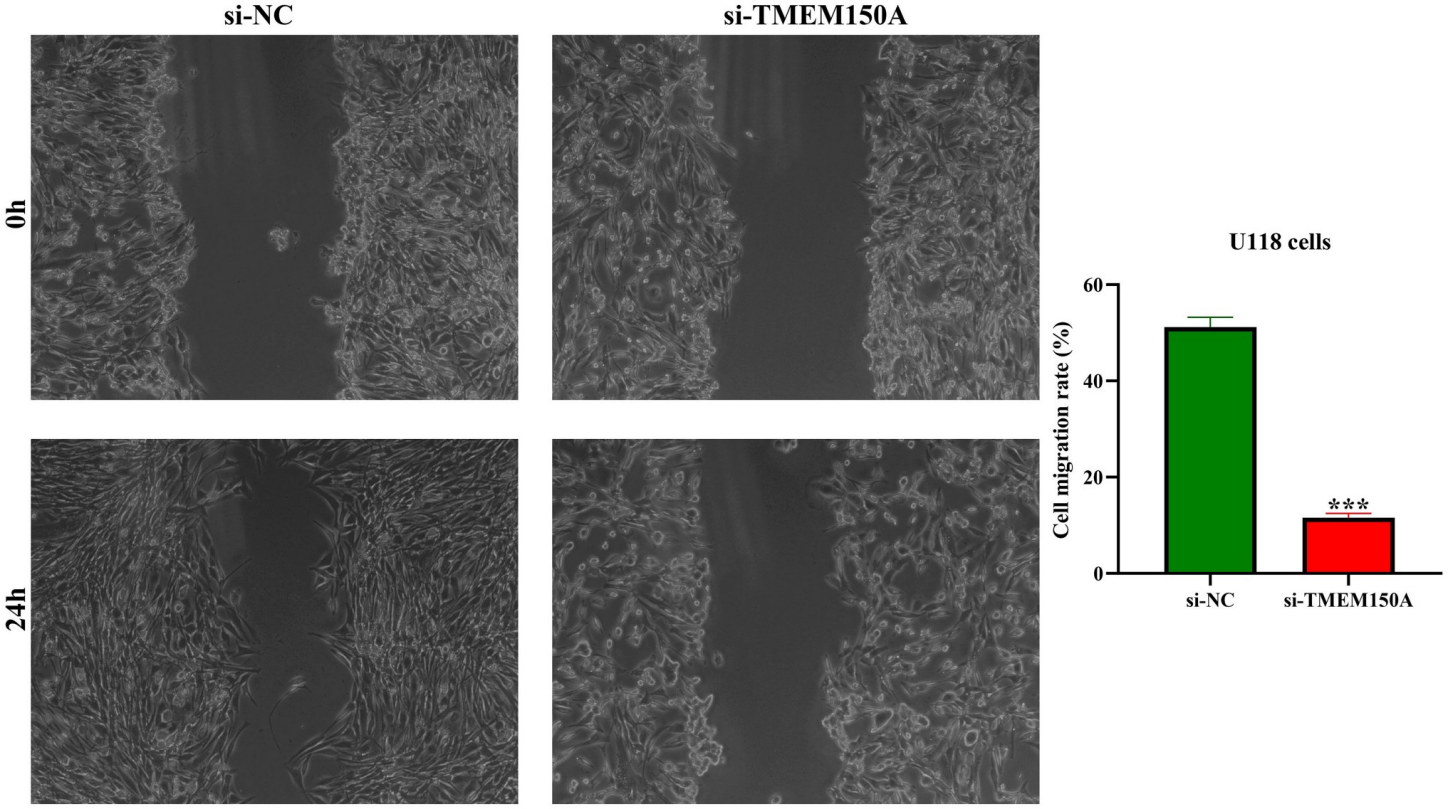
Decreased expression of TMEM150A inhibits cell migration in GBM using wound healing. Note: TMEM, transmembrane protein.

The efficacy of the cell model for inhibiting *TMEM150A* expression was confirmed through RT-PCR and Western blotting (Fig 7A and 7B). CCK-8 revealed that inhibiting *TMEM150A* expression could delay GBM cell proliferation (Fig 7C). Furthermore, assessments conducted via wound healing and Transwell experiments demonstrated that inhibiting *TMEM150A* expression could inhibit GBM cell migration and invasion (Figs 7D–7F and 8).

### *TMEM150A* overexpression was associated with the tumour immune microenvironment

**Fig 9:**
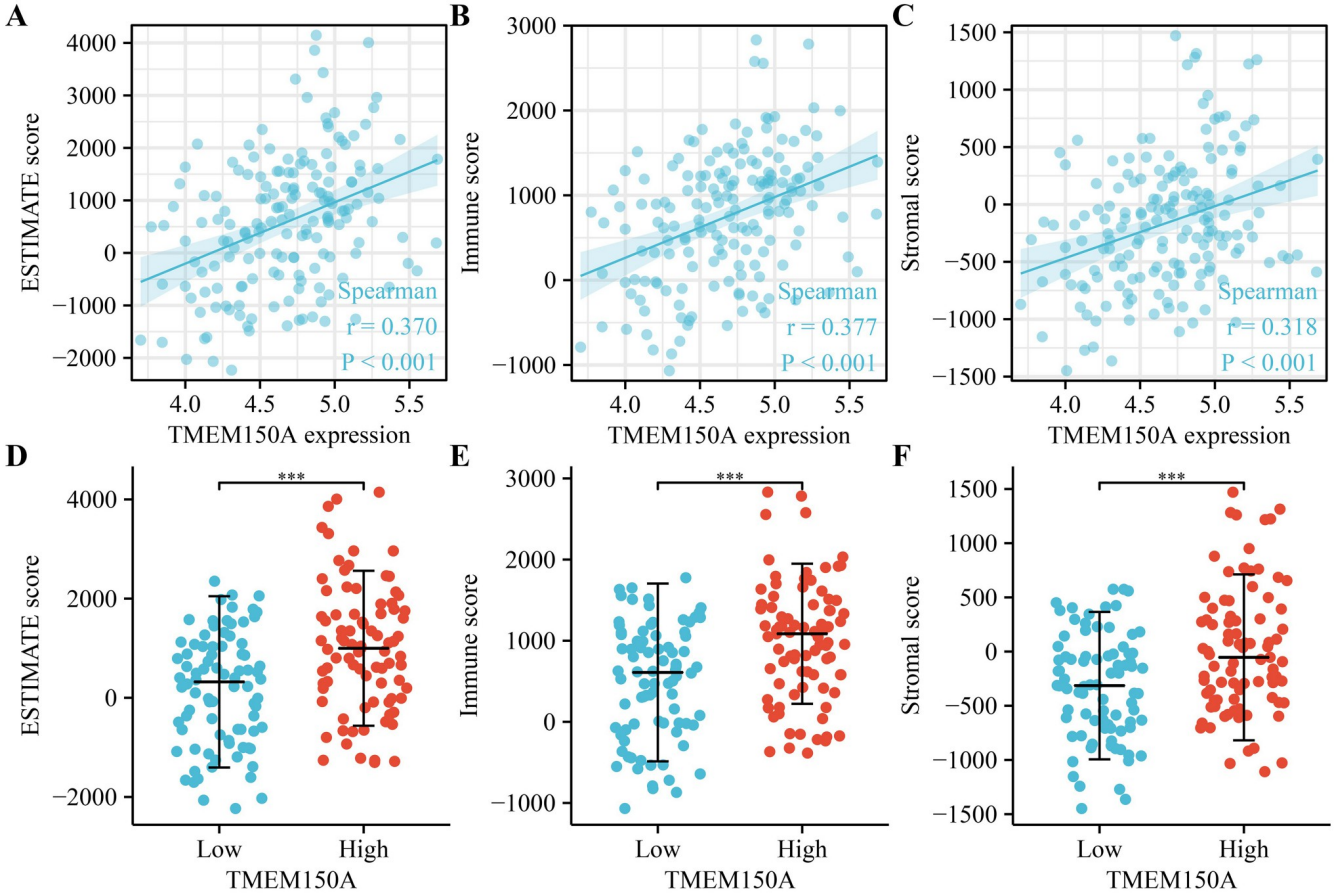
TMEM150A expression was associated with the immune status in GBM. Note: TMEM, transmembrane protein; GBM, glioblastoma multiforme.

**Fig 10:**
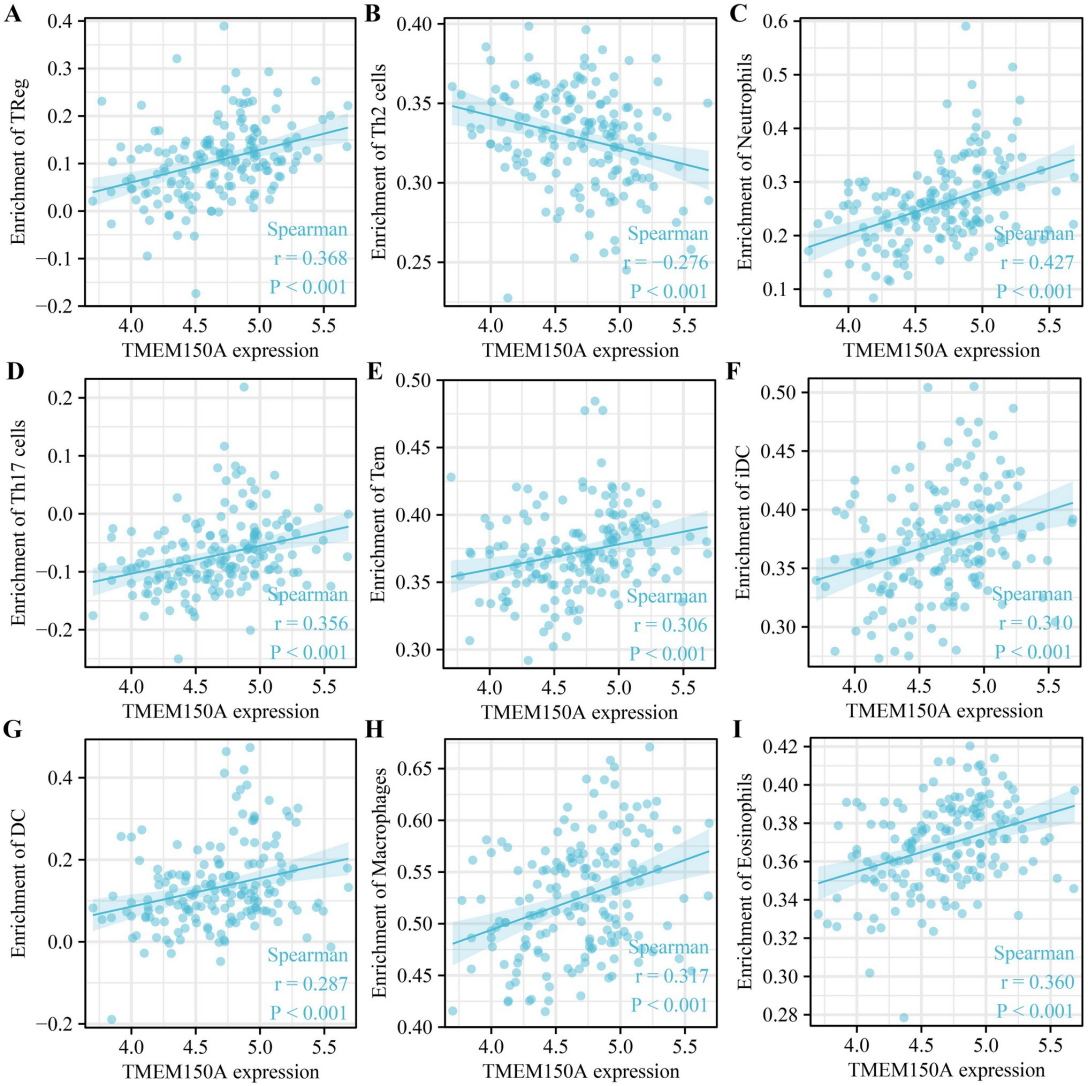
TMEM150A expression was associated with immune cells in GBM using TCGA. Note: TMEM, transmembrane protein; GBM, glioblastoma multiforme; TCGA, The Cancer Genome Atlas.

**Fig 11:**
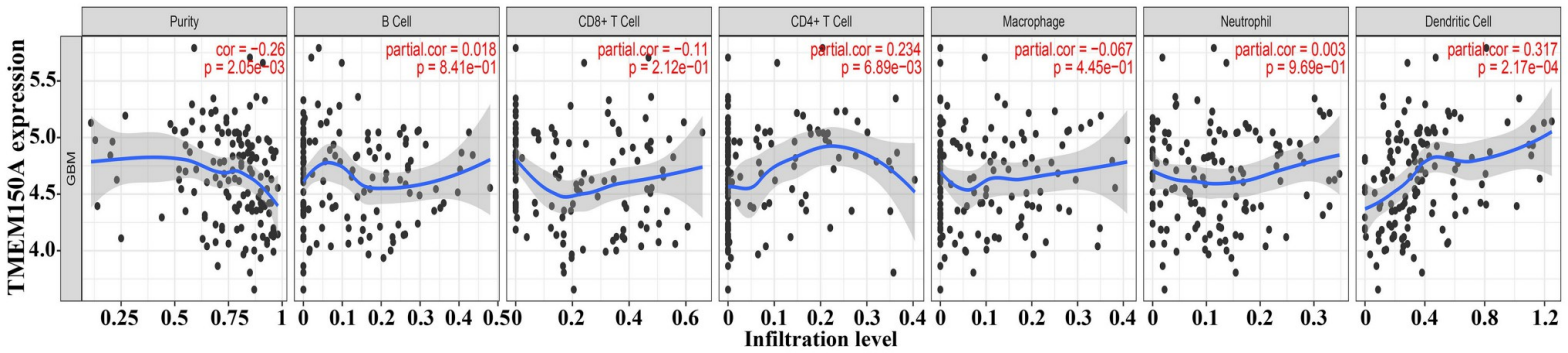
TMEM150A expression was associated with tumour purity and GBM immune cells in TIMER. Note: TMEM, transmembrane protein; GBM, glioblastoma multiforme; TIMER, Tumour IMmune Estimation Resource.

Increased *TMEM150A* expression within GBM tissues sourced from TCGA was positively correlated with immune, estimate, and stromal scores (Fig 9A–9C). Significant differences were observed between groups with *TMEM150A* overexpression and low expression (Fig 9D–9F). *TMEM150A* overexpression was found to be associated with various immune cell types such as T helper (Th) 1 cells, natural killer (NK) cells, NK cells expressing cluster of differentiation (CD) 56 surface antigen in low density (NK CD56dim cells), cytotoxic cells, T cells, activated dendritic cells (aDCs), dendritic cells (DCs), eosinophils, interstitial DCs (iDCs), macrophages, neutrophils, memory T cells (Tems), Th17 cells, Th2 cells, and regulatory T cells (Tregs) (Fig 10). Moreover, the levels of 24 immune cell types between *TMEM150A* overexpression and low-*TMEM150A* expression groups in the patients with GBM were showed (S3 Fig). Additionally, the TIMER database revealed a significant correlation between *TMEM150A* overexpression and the levels of tumour purity, CD4^+^ T cells, and DCs (Fig 11).

### *TMEM150A* overexpression was significantly correlated with immune cell markers

**Fig 12:**
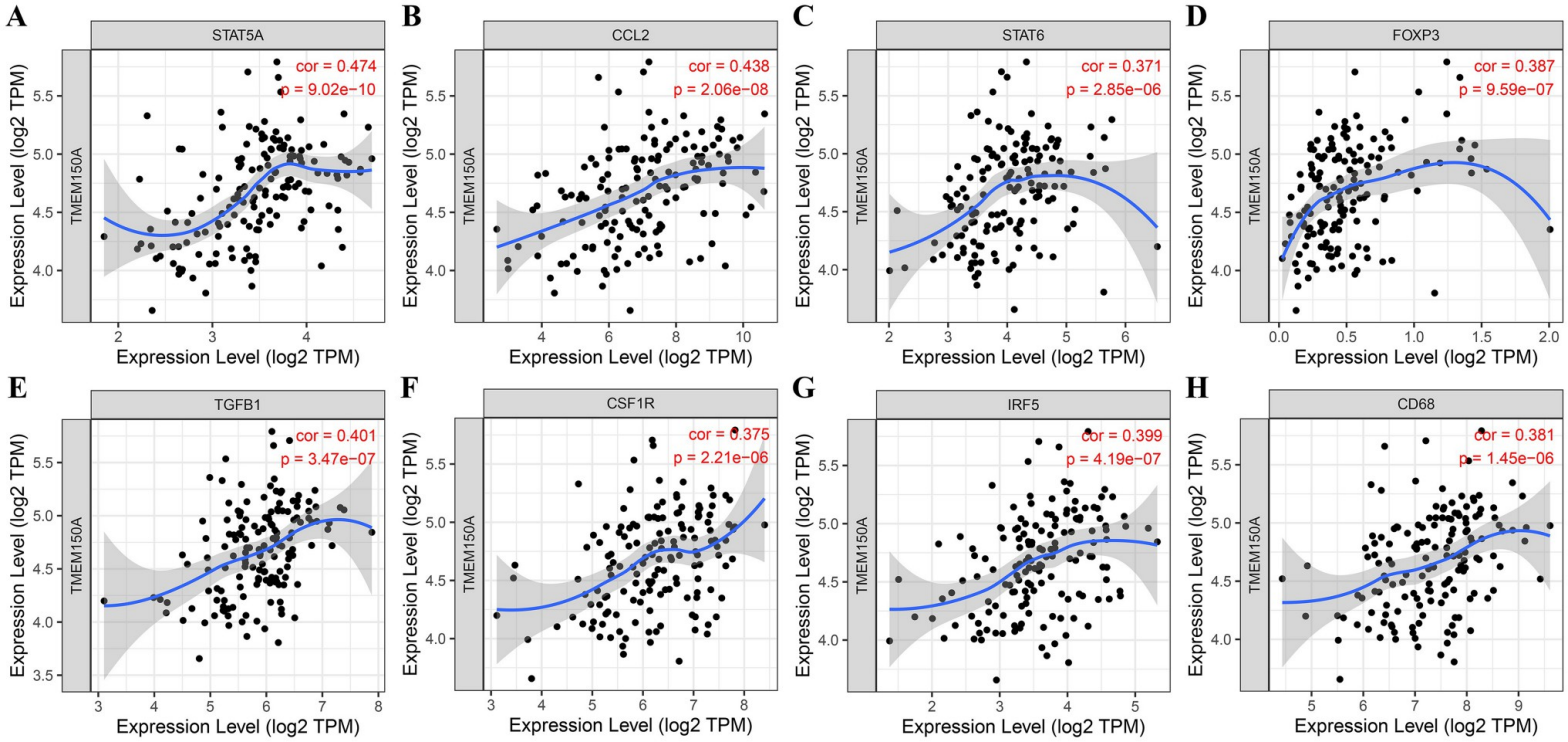
TMEM150A expression was associated with GBM immune cell markers in TIMER. Note: TMEM, transmembrane protein; GBM, glioblastoma multiforme; TIMER, Tumour Immune Estimation Resource.

**Fig 13:**
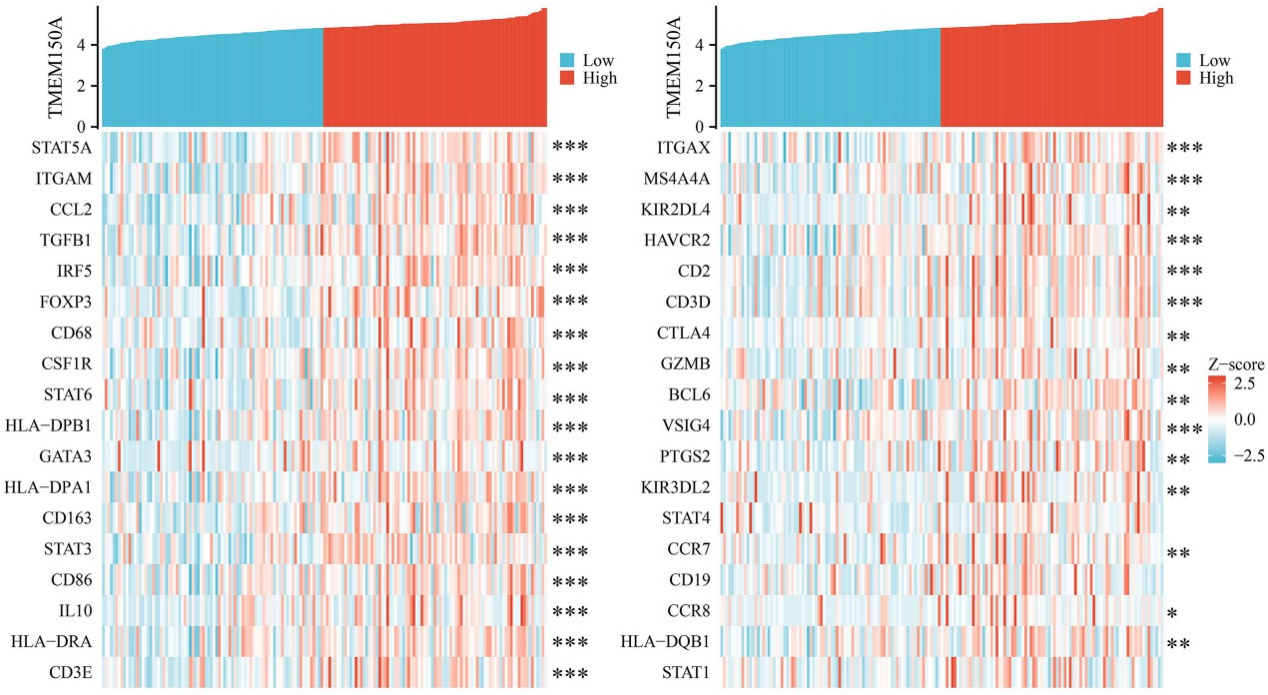
TMEM150A expression was associated with GBM immune cell markers in TCGA. Note: TMEM, transmembrane protein; GBM, glioblastoma multiforme; TCGA, The Cancer Genome Atlas.

Results from the TIMER database demonstrated that *TMEM150A* overexpression was significantly correlated with immune cell markers, including signal transducer and activator of transcription (*STAT*) *5A*, integrin alpha M chain (*ITGAM*), chemokine ligand 2 (*CCL2*), transforming growth factor beta 1 (*TGFB1*), interferon regulatory factor 5 (*IRF5*), forkhead box protein 3 gene (*FOXP3*), *CD68*, colony stimulating factor 1 receptor (*CSF1R*), *STAT6*, human leukocyte antigen (*HLA*)-class II histocompatibility antigen, DP(W2) beta chain (*DPB1*), GATA binding protein 3 (*GATA3*), *HLA-*class II DP alpha 1 (*DPA1*), *CD163*, *STAT3*, *CD86*, interleukin (IL)-10, *HLA-*class II histocompatibility antigen, DR alpha chain (*DRA*), CD3*-*epsilon *CD-3E*, integrin subunit alpha X (*ITGAX*), membrane spanning 4-domains A4A (*MS4A4A*), killer cell immunoglobulin like receptor, two immunoglobulin domains and long cytoplasmic tail 4 (*KIR2DL4*), hepatitis A virus cellular receptor 2 (*HAVCR2*), *CD2*, CD3-delta (*CD3D*), and cytotoxic T-lymphocyte-associated protein 4 (*CTLA4*) (Fig 12 and Table 5). Moreover, TCGA verified the relationship between most immune cell markers and *TMEM150A* levels, as indicated in Fig 13.

**Table 5:** TMEM150A expression was associated with GBM cell markers in the TIMER database.

Markers	Cor	P
STAT5A	0.474210258	9.02E-10
ITGAM	0.447512498	9.72E-09
CCL2	0.438365298	2.06E-08
TGFB1	0.401350971	3.47E-07
IRF5	0.398720732	4.19E-07
FOXP3	0.386906437	9.59E-07
CD68	0.38083846	1.45E-06
CSF1R	0.374509134	2.21E-06
STAT6	0.370632464	2.85E-06
HLA-DPB1	0.366243148	3.79E-06
GATA3	0.364936711	3.52E-06
HLA-DPA1	0.335085709	2.56E-05
CD163	0.323770657	4.89E-05
STAT3	0.312334982	9.18E-05
CD86	0.309322772	0.000107915
IL10	0.307058009	0.000113091
HLA-DRA	0.306390977	0.000126114
CD3E	0.301066838	0.000166701
ITGAX	0.291051827	0.000277902
MS4A4A	0.28283275	0.000417141
KIR2DL4	0.276280632	0.0005464
HAVCR2	0.27002332	0.000767399
CD2	0.266391245	0.000907538
CD3D	0.263283483	0.001008514
CTLA4	0.259312374	0.001208938
GZMB	0.254419927	0.001505702
BCL6	0.251082251	0.001795947
VSIG4	0.243838205	0.002447221
PTGS2	0.242836369	0.002552488
KIR3DL2	0.236533159	0.003243681
STAT4	0.210027073	0.009275964
CCR7	0.193736346	0.016538728
CD19	0.188578766	0.019572342
CCR8	0.186847867	0.020742863
HLA-DQB1	0.171327383	0.034328901
STAT1	0.159399166	0.049143464

Note: TMEM, transmembrane protein; GBM, glioblastoma multiforme; TIMER, Tumour IMmune Estimation Resource.

### *TMEM150A* overexpression was associated with RNA modifications

**Fig 14:**
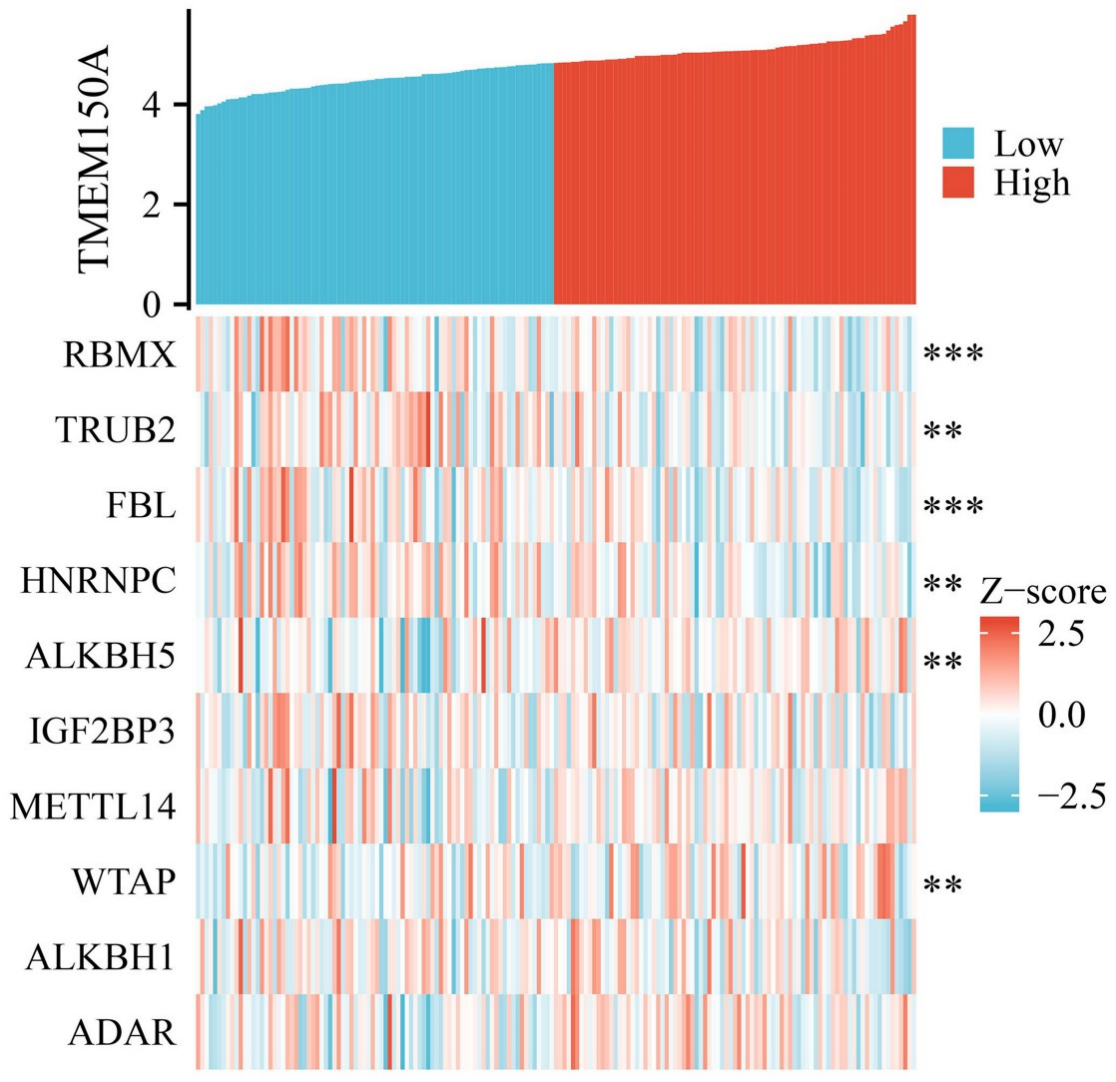
TMEM150A expression was associated with the RNA modification genes of GBM in TCGA. Note: TMEM, transmembrane protein; RNA, ribonucleic acid; GBM, glioblastoma multiforme; TCGA, The Cancer Genome Atlas.

The RM2Target database revealed several RNA modification factors that were associated with *TMEM150A*, including alkB homolog (*ALKBH*) *5*, *METTL16*, *METTL14*, Wilms tumour 1 associated protein (*WTAP*), adenosine deaminase, adenosine deaminase RNA specific B1, zinc finger CCHC-type containing 4, fat mass and obesity-associated protein, vir-like m6A methyltransferase associate, nucleolar protein 58, heterogeneous nuclear ribonucleoproteins A2/B1, insulin-like growth factor 2 mRNA-binding protein (*IGF2BP) 1*, YTH N6-methyladenosine RNA binding protein C1, *METTL3*, zinc finger CCCH-type containing 13, WD repeat domain 4, *METTL5*, *METTL1*, YTH N6-methyladenosine RNA binding protein 2, *ALKBH1*, *IGF2BP3*, THO complex subunit 4, heterogeneous nuclear ribonucleoproteins C1/C2 (*HNRNPC*), fibrillarin (*FBL*), TruB pseudouridine synthase family member 2 (*TRUB2*), and RNA binding motif protein X-linked (*RBMX*). Fig 14 and Table 6 reveal that *TMEM150A* expression levels were correlated with *RBMX*, *TRUB2*, *FBL*, *HNRNPC*, *ALKBH5*, and *WTAP* in TIMER and TCGA databases.

**Table 6:** TMEM150A expression was associated with RNA modifications of GBM in the TIMER database.

Gene	Cor	P
ALKBH5	0.327171538	4.04E-05
METT10D	0.245248817	0.002305679
METTL14	0.193176792	0.016858428
WTAP	0.175736949	0.029790943
ADAR	0.170787932	0.034905916
ADARB1	0.154011365	0.057403529
ZCCHC4	0.104050233	0.200330021
FTO	0.053006849	0.514797858
KIAA1429	0.019249327	0.813101356
NOP58	-0.017121681	0.833442001
HNRNPA2B1	-0.02793414	0.731498583
IGF2BP1	-0.028988371	0.722064809
YTHDC1	-0.036300645	0.655629531
METTL3	-0.043474328	0.593240358
ZC3H13	-0.04978355	0.540712813
WDR4	-0.058257274	0.473999156
METTL5	-0.065380698	0.421586685
METTL1	-0.13600512	0.09366591
YTHDF2	-0.138678917	0.087345835
ALKBH1	-0.174771823	0.030832093
IGF2BP3	-0.196098535	0.015246626
THOC4	-0.235277365	0.003488882
HNRNPC	-0.334053997	2.44E-05
FBL	-0.339220377	2.01E-05
TRUB2	-0.342497286	1.66E-05
RBMX	-0.393091686	6.24E-07

Note: TMEM, transmembrane; RNA, ribonucleic acid; GBM, glioblastoma multiforme; TIMER, Tumour IMmune Estimation Resource.

### *TMEM150A* was involved in the signalling mechanisms of GBM progression

GSEA revealed that *TMEM150A* was associated with several key signalling pathways such as *IL-17*, tumour necrosis factor (*TNF*), nuclear factor-kappa B (*NF-κB*), nucleotide oligomerisation domain-like receptor, chemokine, Toll-like receptor, Th17 cell differentiation, cell cycle, B cell receptor, Janus kinase/STAT (JAK/STAT), cyclic adenosine monophosphate (cAMP), Th1 and Th2 cell differentiation, ferroptosis, deoxyribonucleotide replication, calcium, and T cell receptor pathways (Table 7).

**Table 7:** Signalling mechanisms involved in TMEM150A expression in GBM.

Description	NES	P
Viral protein interaction with cytokine and cytokine receptor	2.918131509	2.65E-09
IL-17 signaling pathway	2.691572812	2.65E-09
Hematopoietic cell lineage	2.700735102	2.65E-09
Rheumatoid arthritis	2.631918951	2.65E-09
Leishmaniasis	2.507757236	2.65E-09
TNF signaling pathway	2.608121523	2.65E-09
NF-kappa B signaling pathway	2.472338151	2.65E-09
Osteoclast differentiation	2.516315394	2.65E-09
Cytokine-cytokine receptor interaction	2.690593331	2.65E-09
NOD-like receptor signaling pathway	2.41667302	2.65E-09
Tuberculosis	2.397307684	2.65E-09
Chemokine signaling pathway	2.365771712	2.65E-09
Phagosome	2.277445328	2.65E-09
Pertussis	2.453274141	4.28E-09
Coronavirus disease—COVID-19	2.109315771	6.66E-09
Lipid and atherosclerosis	2.092986955	1.54E-08
Staphylococcus aureus infection	2.360198294	2.84E-08
Toll-like receptor signaling pathway	2.308287324	4.07E-08
Th17 cell differentiation	2.274581192	1.01E-07
Inflammatory bowel disease	2.408088779	1.35E-07
Influenza A	2.16671301	1.53E-07
Legionellosis	2.357755156	3.94E-07
Asthma	2.242844292	1.34E-06
Neuroactive ligand-receptor interaction	-1.897368767	1.74E-06
Allograft rejection	2.249788516	1.85E-06
Cell cycle	-2.044202048	1.85E-06
Neutrophil extracellular trap formation	2.116953929	2.37E-06
Synaptic vesicle cycle	-2.139161712	4.58E-06
Toxoplasmosis	2.033618751	1.04E-05
Intestinal immune network for IgA production	2.227591328	1.04E-05
Complement and coagulation cascades	2.116809353	2.34E-05
Systemic lupus erythematosus	2.139565241	5.32E-05
C-type lectin receptor signaling pathway	2.025220109	5.32E-05
B cell receptor signaling pathway	1.99992968	6.93E-05
Malaria	2.096515564	8.85E-05
Cholesterol metabolism	2.076531232	0.000119109
Necroptosis	1.877213863	0.00020778
Type I diabetes mellitus	2.075710698	0.000240009
JAK-STAT signaling pathway	1.838299559	0.000240009
Graft-versus-host disease	2.053999416	0.000320573
Kaposi sarcoma-associated herpesvirus infection	1.756135432	0.000374
Amoebiasis	1.831397893	0.00071861
Arachidonic acid metabolism	2.035296477	0.00071861
Salmonella infection	1.64750994	0.000916268
Nicotine addiction	-1.943873936	0.000953128
Glutamatergic synapse	-1.792271003	0.00095578
GABAergic synapse	-1.810799653	0.001002667
Epstein-Barr virus infection	1.633550379	0.001002673
Retrograde endocannabinoid signaling	-1.703743542	0.001051722
cAMP signaling pathway	-1.63403988	0.001402037
Yersinia infection	1.739903151	0.00155358
Morphine addiction	-1.757092293	0.00179734
Chagas disease	1.807735773	0.002006058
Primary immunodeficiency	1.902723023	0.002982821
Th1 and Th2 cell differentiation	1.801444316	0.003156869
Shigellosis	1.570944177	0.003671997
Pathogenic Escherichia coli infection	1.552440012	0.003698618
Fluid shear stress and atherosclerosis	1.664620804	0.004758112
Lysosome	1.651441043	0.004867696
Epithelial cell signaling in Helicobacter pylori infection	1.76135419	0.005133051
MicroRNAs in cancer	-1.606615384	0.005488737
Insulin secretion	-1.700088671	0.005488737
Cytosolic DNA-sensing pathway	1.840016283	0.006573315
Fc epsilon RI signaling pathway	1.728539303	0.006989543
Chemical carcinogenesis—DNA adducts	1.807477135	0.007266373
Human T-cell leukemia virus 1 infection	1.482135158	0.009919705
Ferroptosis	1.75844298	0.01045654
Cholinergic synapse	-1.615373511	0.01143614
Leukocyte transendothelial migration	1.637821436	0.012136711
alpha-Linolenic acid metabolism	1.75619479	0.012660616
Fanconi anemia pathway	-1.669390129	0.013806105
Regulation of lipolysis in adipocytes	-1.711142378	0.013948814
Cell adhesion molecules	1.536562471	0.016724539
Antigen processing and presentation	1.682618797	0.016724539
Dopaminergic synapse	-1.519581617	0.01732848
Axon guidance	-1.449983659	0.01732848
DNA replication	-1.759069927	0.01732848
Taste transduction	-1.599031028	0.018329191
Histidine metabolism	1.74684501	0.018329191
Spliceosome	-1.517631277	0.018785267
Chemical carcinogenesis—reactive oxygen species	1.423267802	0.021412104
Measles	1.533851005	0.022087845
Viral myocarditis	1.657168464	0.022204176
Antifolate resistance	1.731641631	0.02242012
Calcium signaling pathway	-1.399632268	0.024309667
Circadian entrainment	-1.531725405	0.030308598
Progesterone-mediated oocyte maturation	-1.514185109	0.030866525
RIG-I-like receptor signaling pathway	1.658796131	0.035614091
Autoimmune thyroid disease	1.674049819	0.03658195
Arrhythmogenic right ventricular cardiomyopathy	-1.484657271	0.040558878
Glutathione metabolism	1.630019787	0.042215687
Cushing syndrome	-1.437641372	0.044865218
Linoleic acid metabolism	1.606399927	0.046362916

Note: TMEM, transmembrane protein; GBM, glioblastoma multiforme.

## Discussion

GBM is characterised by its high malignancy and frequent recurrence, even after surgery, resulting in poor patient prognosis. The survival duration of patients with GBM depends on various factors, with less than 20% surviving beyond 2 years with surgery-based comprehensive treatment. Targeted therapies have shown promise in treating these cancer types [21]. Several studies have demonstrated a significant correlation between glioblastoma progression and transmembrane proteins [5–8]. For instance, *TMEM230* expression levels are crucial in preserving normal vascular structural integrity and promoting vascular network formation. Conversely, *TMEM230* downregulation could result in compromised cell migration, matrix adhesion, and delivery in GBM. Increased *TMEM230* levels might promote cell migration, extracellular scaffold remodelling, vascular hyperplasia, and abnormal vascular formation in GBM. Targeting *TMEM230* could inhibit GBM cell proliferation, tumour-driven angiogenesis, and antiangiogenic therapies [5]. In contrast, decreased *TMEM150A* expression could result in LPS-induced cytokine secretion, indicating that *TMEM150A* could play a crucial role in cell homeostasis [9]. However, its implications within GBM have not been revealed. Our study has uncovered that *TMEM150A* expression was significantly elevated in GBM and serves as a diagnostic marker. *TMEM150A* overexpression was associated with poor OS, DFS, DSS, and PFI in patients with GBM, which was consistent in the subgroup analysis. Cox analysis revealed that *TMEM150A* overexpression was an independent risk factor for poor survival in patients with GBM. Furthermore, prognostic nomograms related to *TMEM150A* expression were associated with the prognosis of patients with cancer. Our findings suggest that *TMEM150A* overexpression could potentially serve as a prognostic biomarker for patients with GBM.

It was well established that *IL-17*, *TNF*, *NF-κB*, Toll-like receptor, cell cycle, JAK-STAT, cAMP, ferroptosis, and others are closely related mechanisms of cancer [22–29]. For instance, Jiang et al. reported that *circKPNB1* was overexpressed in GBM and was correlated with poor survival time. Their findings revealed that the *TNF-α*/*NF-κB* signalling mechanism resulted in circKPNB1 overexpression, consequently promoting the viability, proliferation, invasion, and stemness of GBM stem cells [23]. Similarly, Zhang et al. reported that the inhibition of COPI coat complex subunit zeta 1 (*COPZ1*) expression inhibited GBM cell proliferation and tumour formation in nude mice. *COPZ1* knockdown resulted in increased nuclear receptor coactivator 4, ferritin degradation, and elevated intracellular ferrous levels, resulting in ferroptosis [8]. It was observed that inhibiting *TMEM150A* expression could delay U118 cell proliferation, migration, and invasion in GBM. Additionally, *TMEM150A* was strongly associated with *IL-17*, *TNF*, *NF-κB*, Toll-like receptor, cell cycle, JAK-STAT, cAMP, ferroptosis, and other pathways. However, our study did not investigate the relationship between *TMEM150A* and these mechanisms, which warrants further exploration in the future.

Diverse strategies exist for treating patients with cancer, and among these, immunotherapy has emerged as a crucial aspect of cancer treatment [30, 31]. This study analysed the relationship between *TMEM150A* overexpression and the immune microenvironment in GBM. Our findings revealed that *TMEM150A* overexpression in GBM tissues was strongly correlated with immune, estimate, and stromal scores, and significantly correlated with tumour purity, Th1 cells, NK cells, NK CD56dim cells, cytotoxic cells, CD4^+^ T cells, T cells, aDCs, DCs, eosinophils, iDCs, macrophages, neutrophils, Tmems, Th17 cells, Th2 cells, and Tregs. Moreover, *TMEM150A* expression levels were correlated with immune cell markers such as *STAT5A*, *ITGAM*, *CCL2*, *TGFB1*, *IRF5*, *FOXP3*, *CD68*, *CSF1R*, *STAT6*, *HLA-DPB1*, *GATA3*, *HLA-DPA1*, *CD163*, *STAT3*, *CD86*, *IL10*, *HLA-DRA*, *CD3-E*, *ITGAX*, *MS4A4A*, *KIR2DL4*, *HAVCR2*, *CD2*, *CD3-D*, *CTLA4*, and others. Additionally, RNA modification genes, such as *RBMX*, *HNRNPC*, *ALKBH5*, and *WTAP*, play a crucial role in cancer biology [32–38]. For instance, the inhibition of *ALKBH5* expression in GSCs significantly down-regulated stem cell survival and invasion after irradiation and mediated the radioresistant properties of stem cells [33]. Similarly, long non-coding RNA SRY-box 2 (SOX2) overlapping transcript (SOX2OT) expression levels were significantly increased in temozolomide (TMZ)-resistant cells and tissue samples, concomitant with GBM progression. Increased SOX2OT levels were closely associated with recurrence risk and poor prognosis. Down-regulating SOX2OT expression could inhibit cell proliferation, promote cell apoptosis, and increase sensitivity to TMZ in GBM. SOX2OT could recruit *ALKBH5* and *ALKBH6* to bind to *SOX2* and demethylate the *SOX2* transcript and subsequent *SOX2* up-regulation, thereby regulating tumour cell apoptosis, cell proliferation, and TMZ resistance [34]. Our study revealed that *TMEM150A* expression levels were significantly correlated with RNA modification genes, namely *RBMX*, *TRUB2*, *FBL*, *HNRNPC*, *ALKBH5*, and *WTAP*, suggesting that *TMEM150A* plays crucial biological roles in GBM progression.

This study revealed that *TMEM150A* plays a crucial role in GBM and offers significant advantages concerning high reliability and large sample sizes in GBM data. However, moving forward, the verification of *TMEM150A* expression in GBM tissues through clinical sampling remains a crucial avenue to explore, thereby substantiating its potential clinical significance. Furthermore, the establishment of cell models designed to inhibit *TMEM150A* expression to investigate its roles and signalling mechanisms involved in GBM cell growth and migration. In summary, *TMEM150A* was overexpressed in GBM tissues and was significantly correlated with diagnosis, poor prognosis, immune status, and RNA modifications in GBM. Inhibition of *TMEM150A* expression could inhibit GBM cell proliferation, migration, and invasion. Thus, TMEM150A overexpression could be a potential biomarker for poor prognosis in patients with GBM.

## Conclusions

*TMEM150A* overexpression was associated with diagnosis, poor prognosis, immune status, and RNA modifications in GBM. Inhibiting *TMEM150A* expression could inhibit GBM cell proliferation, migration, and invasion. *TMEM150A* might serve as a biomarker of poor prognosis in patients with GBM.

## Supporting information

S1 Fig Elevated TMEM150A expression was associated with short OS and DFS in GBM patients. Note: TMEM, transmembrane protein; OS, overall survival; DFS, disease-free survival; GBM, glioblastoma multiforme.
(TIF)

S2 Fig TMEM150A correlating the nomogram in terms of the DSS and PFI of patients with GBM. Note: TMEM, transmembrane protein; DSS, disease-specific survival; PFI, progression-free interval; GBM, glioblastoma multiforme.
(TIF)

S3 Fig The immune cell levels in high- and low-expression TMEM150A groups. Note: TMEM, transmembrane protein.
(TIF)
