# Supplementary figures and images for: Overexpression of TMEM150A in glioblastoma multiforme patients correlated with dismal prognoses and compromised immune statuses
Source: PLoS One. 2023 Dec 6;18(12):e0294144. doi: 10.1371/journal.pone.0294144 (PMC10699650; doi:10.1371/journal.pone.0294144)

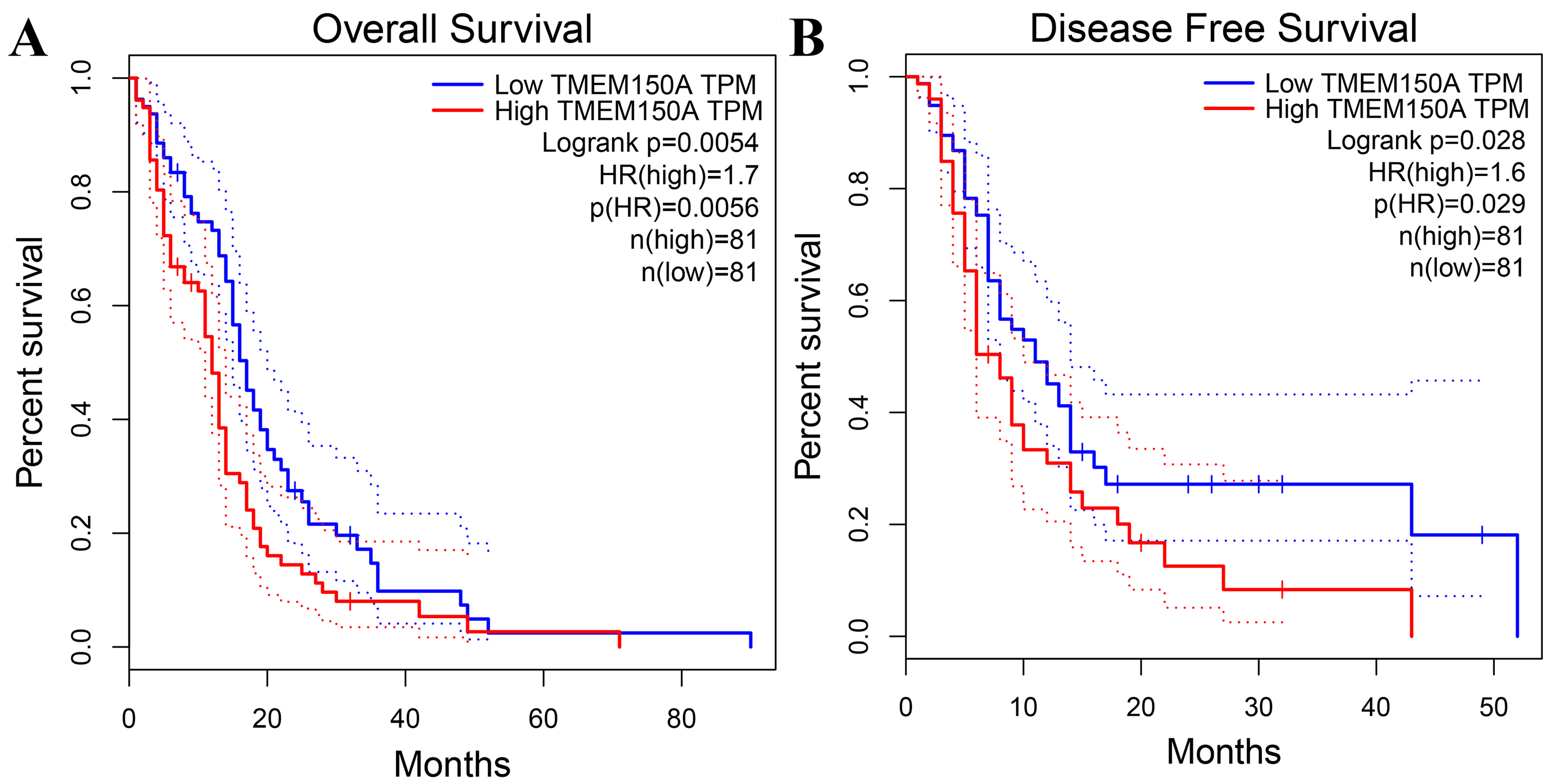

Supplement: S1 Fig — Note: TMEM, transmembrane protein; OS, overall survival; DFS, disease-free survival; GBM, glioblastoma multiforme. (TIF) [file pone.0294144.s001.tif]

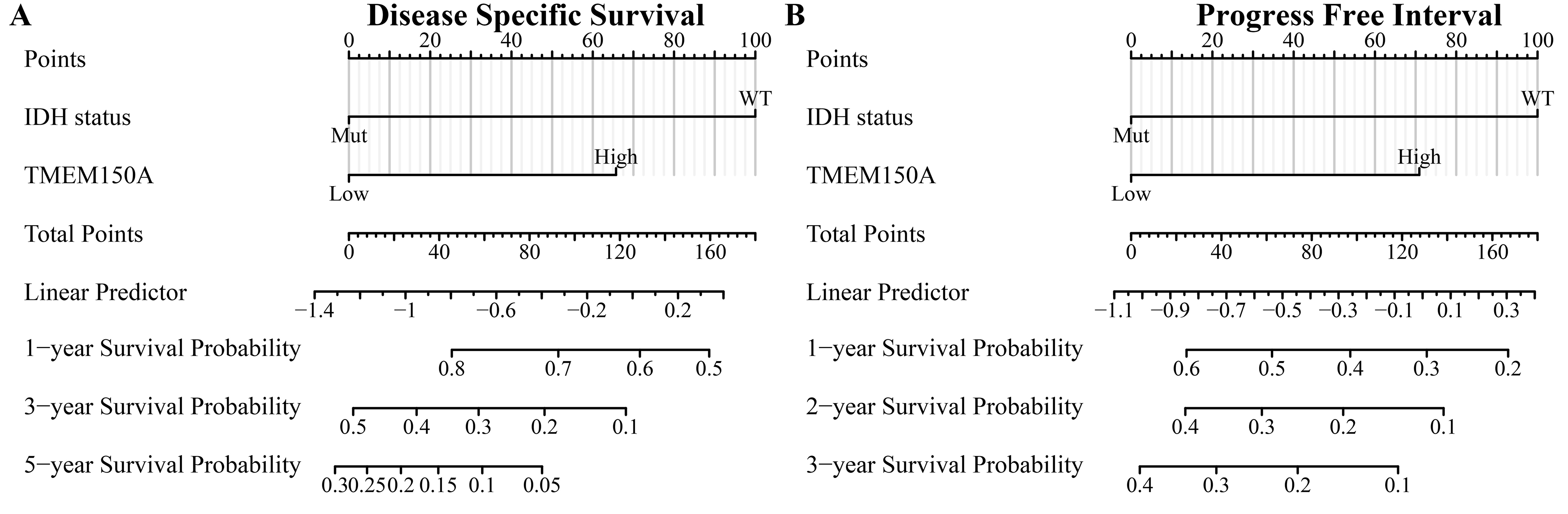

Supplement: S2 Fig — Note: TMEM, transmembrane protein; DSS, disease-specific survival; PFI, progression-free interval; GBM, glioblastoma multiforme. (TIF) [file pone.0294144.s002.tif]

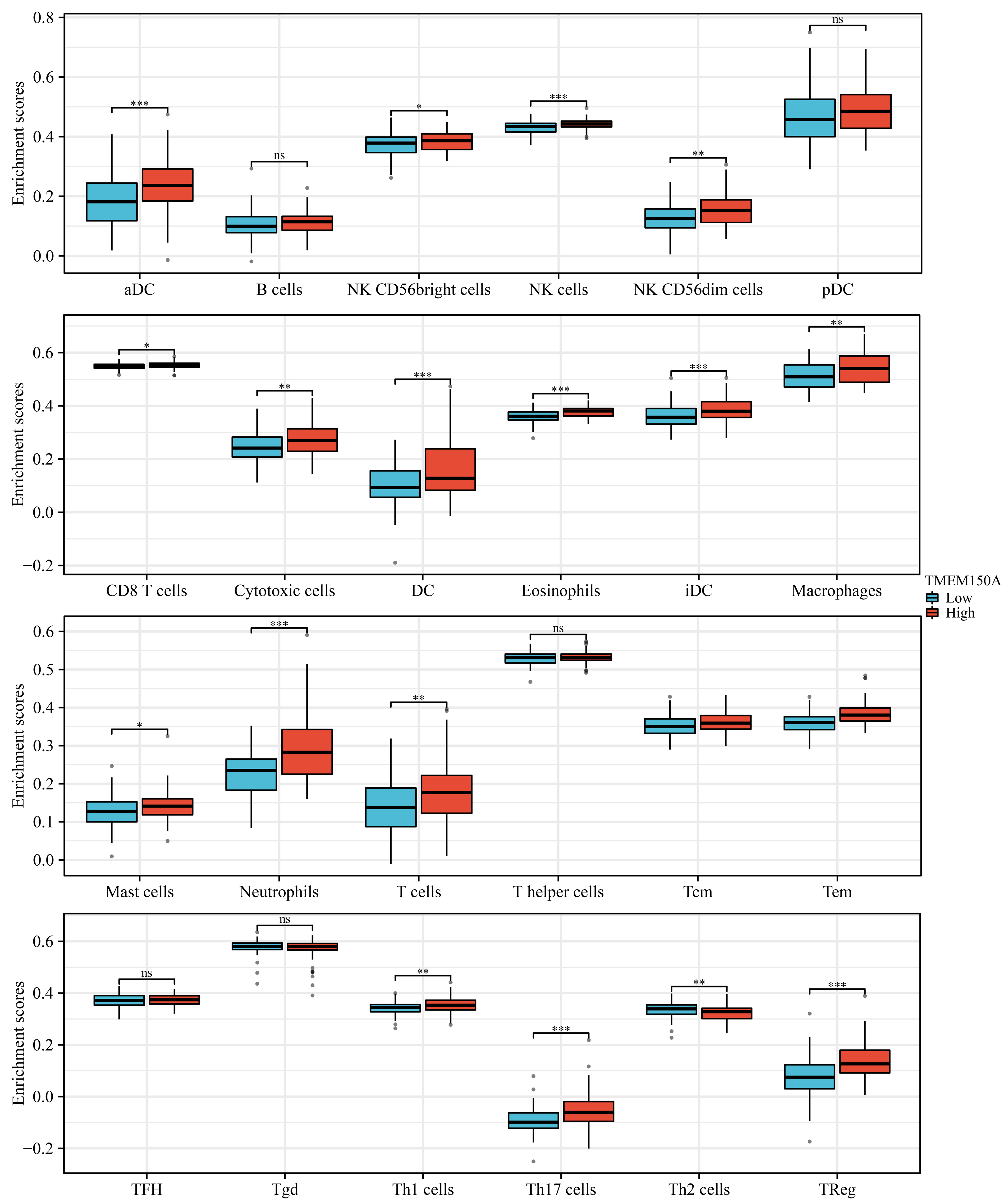

Supplement: S3 Fig — Note: TMEM, transmembrane protein. (TIF) [file pone.0294144.s003.tif]
